# Biodegradable and Multifunctional Polymer Micro-Tubes for Targeting Photothermal Therapy

**DOI:** 10.3390/ijms150711730

**Published:** 2014-07-02

**Authors:** Xin Wang, Guoping Yu, Xiyu Han, Hua Zhang, Jing Ren, Xia Wu, Yanfeng Qu

**Affiliations:** 1 Food Science College, Northeast Agricultural University, 59 GongBin Road, XiangFang District, Harbin 150030, China; E-Mails: neauwangxin@gmail.com (X.W.); renjing1979@126.com (J.R.); wuxiatt@126.com (X.W.); Yanfengqu@gmail.com (Y.Q.); 2 School of Clinical Medical, Heilongjiang University of Chinese Medicine, Harbin 150040, China; E-Mail: xiyuhan@gmail.com; 3 Heilongjiang International Travel Healthcare Center, 9 GanShui Road, Harbin 150036, China; 4 School of Food Science and Engineering, Harbin Institute of Technology, 73 HuangHe Road, NanGang District, Harbin 150090, China; E-Mail: zhhua@hit.edu.cn

**Keywords:** micro-tube, soybean protein isolate, magnetic manipulation, photothermal therapy

## Abstract

We describe an innovative form of polymer micro-tubes with diverse functions including biodegradation, magnetic manipulation, and photothermal effect that employs and activates photothermal therapy to target cancer cells. The micro-tube comprised soybean protein isolate, poly-l-glutamic acid, magnetite nanoparticles, plus gold nanoparticles. Through electrostatic force, these components, with opposite charges, formed pairs of layers in the pores of the template, various bilayers of soybean protein isolate and poly-l-glutamic acid served as the biodegradable building wall to each micro-tube. The layers of magnetite nanoparticle functionalized micro-tubes enabled the micro-tube manipulate to target the cancer cells by using an external magnetic field. The photo-thermal effect of the layer of gold nanoparticles on the outer surface of the micro-tubes, when under irradiation and when brought about by the near infrared radiation, elevated each sample’s temperature. In addition, and when under the exposure of the near infrared radiation, the elevated temperature of the suspension of the micro-tubes, likewise with a concentration of 0.2 mg/mL, and similarly with a power of 2 W and as well maintained for 10 min, elevated the temperature of the suspension beyond 42 °C. Such temperatures induced apoptosis of target cancer cells through the effect of photothermal therapy. The findings assert that structured micro-tubes have a promising application as a photothermal agent. From this assertion, the implications are that this multifunctional agent will significantly improve the methodology for cancer diagnosis and therapy.

## 1. Introduction

Photothermal therapy offers a promising treatment for premalignant and early-stage cancer due to its minimal and diffusive nature and deep tissue penetration [1,2]. The combination of near infrared radiation (NIR), and a corresponding photothermal agent, was used to perform physical therapy to destroy cancer cells [3]. When treated by NIR, the photothermal agent (PTA) converted the light energy to a heat source and then rapidly increased the local temperature. The elevated temperature in the vicinity of the PDA then triggered apoptosis within the cancer cell [4].

Among the diverse range of photothermal agents, gold nanoparticles (Au NPs) are used in biological and medical applications because of their beneficial biocompatibility and optical properties [5]. By controlling the size and geometry of the Au NPs, the wavelength of plasmon resonance of the Au NPs were tuned to the region of the NIR with minimal absorption and effective penetration when related to human tissue. However, in relation to photothermal therapy, a requirement is that the PTAs are located. However, in spite of this requirement in relation to photothermal therapy, and before conducting the therapy, another requirement is that the PTAs are located to the target cancer cells. 

In addition, to avoid the adverse effect on normal cells, degradation of the PTAs was required after the treatment of the NIR. Likewise, the inherent drawback of the PTAs limited the application of photothermal therapy. Hence, the photothermal therapy offer challenges to develop degradable PTAs with a controllable location to human tissue. To address this problem the experiment employed a form of polymer micro-tubes with diverse functions including biodegradation, magnetic manipulation, and photothermal effect; the experiment then employed and activated photothermal effect to target cancer cells. 

With regard to photothermal agents, most past research efforts, found within the applicable research literature, have focused on spherical particles, such as porphyrin bilayer cerasomes [6], together with micro-capsules [7]. In this experiment, the tubular structure of photothermal agents provided an opportunity to develop multifunctional micro-tubes by the application of inner and outer surfaces [8]. In conjunction with a micro-porous template, the construction of the polymer micro-tube is based on the electrostatic Layer-by-Layer (LbL) technique [9]. To achieve this outcome, two compositions of micro-tube soybean protein isolate (SPI) and poly-l-glutamic acid (PGA) were alternately deposited on the pores of the template through electrostatic interaction to form biodegradable building walls on each micro-tube [10].

The Fe_3_O_4_ NPs and the Au NPs were correspondingly assembled to the inner and outer surfaces of the micro-tubes. By applying the external magnetic field, the micro-tubes containing the Fe_3_O_4_ NPs enabled magnetic manipulation, which were then directed to the targeted tissue location [11]. To enhance the performance of photothermal therapy, the assembly process causes the aggregation of Au NPs on the microtube, which allows for strong absorption in the NIR region. In this case, such multifunctional polymer micro-tubes, by remote control, provide a method to target cancer cell photo-thermal therapy. In addition, the findings also offer new candidates promising treatment for premalignant and early-stage cancer by offering photothermal therapy in this new practical way.

## 2. Results and Discussion

### 2.1. Fabrication of the Multifunctional Polymer Micro-Tubes

**Figure 1:**
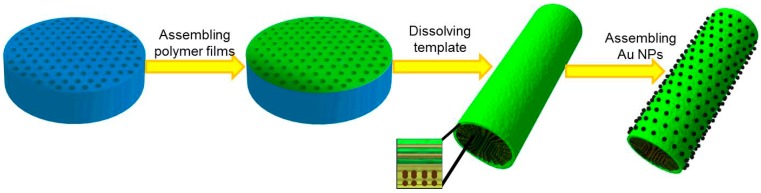
Schematic illustration of the fabrication of Au NPs-(PGA/SPI)_14.5_-Fe_3_O_4_ NPs micro-tubes. The blue porous pie represents the template for deposition of components. The brown dots and the colorful bands inside the micro-tube represent magnetic nanoparticles and (poly-l-glutamic acid) (PGA)/soybean protein isolate (SPI) multilayers, and the black dots on the outer surfaces of the micro-tubes represent the Au NPs.

Depicted in Figure 1, and summarized in three steps, is the process of fabricating the multifunctional polymer micro-tubes that include: (1) The preparation of the polymer micro-tubes; (2) The releasing of the micro-tubes from the template; (3) The assembly of Au NPs on the outer surface of the micro-tube.

The Au NPs, with a size of 2–5 nm and the Fe_3_O_4_ NPs, with average diameter of 15 nm, were synthesized according to the reported methods [12,13] The preparation of the micro-tubes, with SPI and PGA, were based on the integration of polymers into the porous template. The positive charged PGA and the negative charged SPI, as a thin film, and one bilayer of (PGA/SPI), were alternately assembled into the pores of the template by immersing the template into each solution. To form the durable polymer building walls of the micro-tubes with PGA on the inner and outer surfaces of the micro-tubes, 14.5 bilayers of (PGA/SPI) thin film was placed into the template pores. Noticing that number of layers are counted by preparation procedure, the successful assembly of the components on the template has been reported [11]. To fabricate the magnetic micro-tubes, the stabilized citrate was then integrated into the pores of the template. The unwanted materials were then removed by polishing the surfaces of the template using a wet cotton swab. 

The micro-tubes, with a positive charged PGA on the outer surface, were then released by resolving the template with CH_2_Cl_2_ and diffusing, independently, the template in both ethanol and water. This procedure was followed by the absorption of the negatively charged Au NPs onto the PGA layer by electrostatic interaction. The resulting Au NPs-(PGA/SPI)_14.5_-Fe_3_O_4_ NPs micro-tubes were then collected by the external magnetic field.

### 2.2. Characterization of the Gold Nanoshelled Micro-Tubes

**Figure 2:**
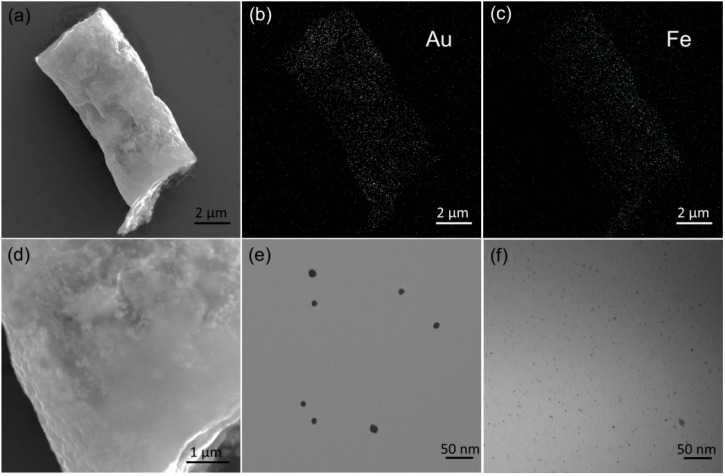
The characterization of the micro-tube. (**a**) SEM image of a single micro-tube; (**b**) The corresponding energy-dispersive X-ray (EDX) mapping images of (**a**) for Au distribution; (**c**) The corresponding EDX mapping images of (**a**) for Fe distribution; (**d**) Enlarged SEM image of the micro-tube; (**e**) TEM images of Fe_3_O_4_ NPs; (**f**) TEM images of Au NPs.

The scanning electronic microscope (SEM) images of a single Au NPs-(PGA/SPI)_14.5_-Fe_3_O_4_ NPs micro-tube are illustrated in Figure 2a. This micro-tube displays a typical cylindrical geometry with length of 10 μm; the length of this micro-tube is in accordance with the thickness of the template. The diameter of the micro-tube was approximately 5 μm, which is in accordance with the pore diameter of the template used. 

The corresponding energy-dispersive X-ray (EDX) spectrum verified the presence of both Au (Figure 2b) and Fe (Figure 2c). This outcome signified the successful assembly of the Fe_3_O_4_ NPs with a dimensional measurement of 10–30 nm (Figure 2e) and the Au NPs with a dimensional measurement of 2–5 nm (Figure 2f). The following enlarged SEM image shows the high coverage of the Au NPs over the micro-tube surface.

### 2.3. Magnetic Manipulation of the Micro-Tubes

**Figure 3:**
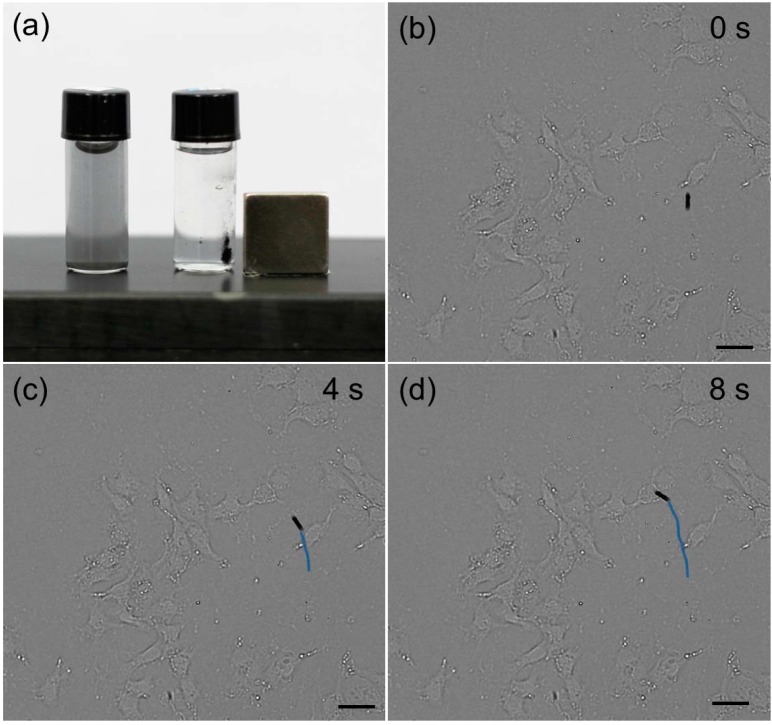
The magnetic manipulation of the Au NPs-(PGA/SPI)_14.5_-Fe_3_O_4_ NPs micro-tubes. (**a**) This photograph showing the aggregation of the Au NPs-(PGA/SPI)_14.5_-Fe_3_O_4_ NPs micro-tubes under the external magnetic; (**b**–**d**) Time-lapse images illustrating the movement of a single micro-tube to the HeLa cells, the blue line shows the trajectory of the locomotion of the micro-tube to the HeLa cells under the external magnetic field. Scale bar = 20 μm.

The outer and inner surfaces of the Au NPs-(PGA/SPI)_14.5_-Fe_3_O_4_ NPs micro-tubes were constructed to have two functions. To conduct the photothermal effect, the first function united the Au NPs on the outer surface of the micro-tubes. Then, to perform the magnetic manipulation of the micro-tubes, the second function assembled the Fe_3_O_4_ NPs on the inner surface of the micro-tubes. Consequently, under the irradiation of the NIR, the Au NPs-(PGA/SPI)_14.5_-Fe_3_O_4_ NPs micro-tubes elevated the temperature through the photo-thermal effect of the Au NPs. In this situation, the micro-tubes were manipulated by using an external magnet (Figure 3a). The micro-tubes were rapidly attracted to the magnet and the black suspension of the micro-tubes became almost colorless. In addition, in the absence of the external magnetic field, the attracted micro-tubes were re-dispersed in a medium. In this way, the micro-tubes were guided to the target cancer cells by using the external magnetic field. It should be noted that the assembly of the Fe_3_O_4_ NPs to the microtube allows for the effective external magnetic manipulation in the compare to bare Fe_3_O_4_ NPs, because of the weak magnetism of a single ultrasmall Fe_3_O_4_ NP.

As shown in Figure 3b–d, the locomotion of a micro-tube in the cell culture occurred over a period of 8 s at 4-s intervals; following this process the micro-tube floated on the surface of the cell culture (Figure 3b). When under the exposure of external magnetic field, the cell culture was located to the HeLa cells (Figure 3b,c); the magnetic micro-tubes were easily manipulated and the magnetic property of the micro-tubes was maintained for more than one month at 4 °C. More importantly, such protocol of microtube also allows for the targeted movement by other physical stimuli (including the light, heat and even osmotic pressure) or the affinity molecules like antibody, aptamer, and target peptide.

### 2.4. Temperature Elevation under Different Concentration of the Micro-Tubes

**Figure 4:**
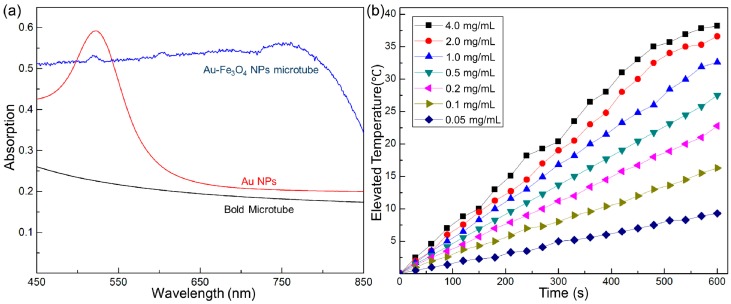
Heating effect of the microtube under the near infrared radiation (NIR) irradiation. (**a**) UV-vis spectrum of the bold microtube (black line), Au NPs (red line), and Au NPs-(PGA/SPI)_14.5_-Fe_3_O_4_ NPs micro-tube (blue line); (**b**) The elevated temperature of suspension of the Au NPs-(PGA/SPI)_14.5_-Fe_3_O_4_ NPs micro-tubes with different concentration (0.05, 0.1, 0.2, 0.5, 1.0, 2.0, 4.0 mg/mL) under the irradiation of NIR conducted every 30 s over 10 min.

Besides the improvement of the magnetic manipulation by integration of Fe_3_O_4_ NPs to the microtube, more importantly, the assembly of Au NPs on the surface of the microtube allow for the absorption wavelength of the in the NIR region, which resolve the key challenges of Au NPs as photothermal agent in cancer therapy. As illustrated in Figure 4a, compare to the maximum wavelength of Au NPs in the visible region (approximately 520 nm), the microtubes assembled Au and Fe_3_O_4_ NPs shows considerable absortion in NIR region. It is the assembly process causes the aggregation of Au NPs on the microtubes, leading the maximum adsorption of the microtubes shift to the NIR region. In this way, under the irradiation of the NIR, the Au NPs on the outer surfaces of the Au NPs-(PGA/SPI)_14.5_-Fe_3_O_4_ NPs micro-tubes converted the light energy into thermal energy. In addition, the photo-thermal effect increased the temperature of the aqueous dispersion of the Au NPs-(PGA/SPI)_14.5_-Fe_3_O_4_ NPs micro-tubes. To investigate the dependence of the elevated temperature on the concentration of the Au NPs-(PGA/SPI)_14.5_-Fe_3_O_4_ NPs micro-tubes; the suspension, with different concentrations of the Au NPs-(PGA/SPI)_14.5_-Fe_3_O_4_ NPs micro-tubes, were irradiated by NIR with a wavelength of 808 nm and a power output of 2 watts. Figure 4 shows the temperature elevation under the exposure of the NIR at 30 s intervals over 10 min. The sample’s temperature was elevated by increasing the period of the NIR. The elevated temperature rose, in the range of 0.05–4 mg/mL, when caused by the concentration within the micro-tubes. When treated for 10 min by NIR, the sample’s temperature, with a concentration of 0.5 mg/mL, exceeded 42 °C. This temperature was enough to induce apoptosis of the cancer cell [14]. In contrast, no substantial change of temperature was observed when the NIR was exposed to distilled water, which indicated the photo-thermal effect of Au NPs-(PGA/SPI)_14.5_-Fe_3_O_4_ NPs, micro-tubes. As a versatile platform of assembling functional components, the microtube also allows for other external physical guidance to the defined location. If needed, the affinity components such as antibody, aptamer and peptides onto the microtube to perform the specific recognition. 

### 2.5. Biodegradable Behavior of the Micro-Tubes

**Figure 5:**
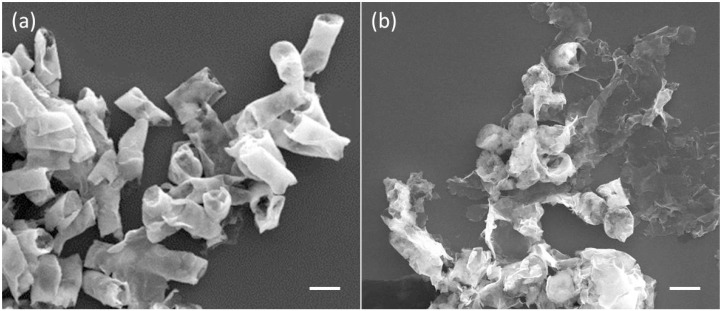
Biodegradable behavior of the Au NPs-(PGA/SPI)_14.5_-Fe_3_O_4_ NPs micro-tubes. (**a**) This photograph shows the SEM images of the micro-tubes before the treatment of chymotrypsin; (**b**) This photograph shows the SEM images of the micro-tubes after the treatment of α-chymotrypsin. Scale bar = 10 μm.

When the micro-tubes were degraded under the treatment of α-chymotrypsin, the Au NPs-(PGA/SPI)_14.5_-Fe_3_O_4_ NPs micro-tubes also showed biodegradable property. The comparison of SEM images of the micro-tubes, before and after the treatment of α-chymotrypsin, verified the biodegradability of the micro-tubes (Figure 5). The SEM image of the enzyme-degraded micro-tubes confirmed the building walls of the micro-tubes became thinner in contrast with the micro-tubes before degrading; in addition, the degraded micro-tubes exhibited a rough surface and collapsed geometry. In the control experiment, a micro-tube was stabilized at 4 °C over one month. This result demonstrates that the assembled Au NPs-(PGA/SPI)_14.5_-Fe_3_O_4_ NPs micro-tubes are biodegradable. The biodegradable behavior of each micro-tube was verified because one of the components of the micro-tube, the soybean protein, was, without difficulty, hydrolyzed by the α-chymotrypsin. The biodegradable property of the micro-tubes extended the application of the micro-tubes especially in the field of biology and medicine.

### 2.6. The Photothermal Effect of the Micro-Tubes to the Viability of HeLa Cells

The Au NPs-(PGA/SPI)_14.5_-Fe_3_O_4_ NPs micro-tubes directed to the HeLa cell, under the irradiation of NIR, induced apoptosis of the HeLa cells through the photothermal effect of the micro-tubes [15]. According to the results in Figure 4a, the photothermal effect of the micro-tubes, when subjected to the radiation of the NIR, heated the cell culture and induced apoptosis of the cells at a temperature far beyond 42 °C. In order to evaluate the photothermal effect of the micro-tubes to the HeLa cells, propidium iodide (PI), a fluorescent cell dye, indicated the apoptosis of the cells by the penetration into the nuclei of the cells through the breakage of membrane of the apoptosis cells.

**Figure 6:**
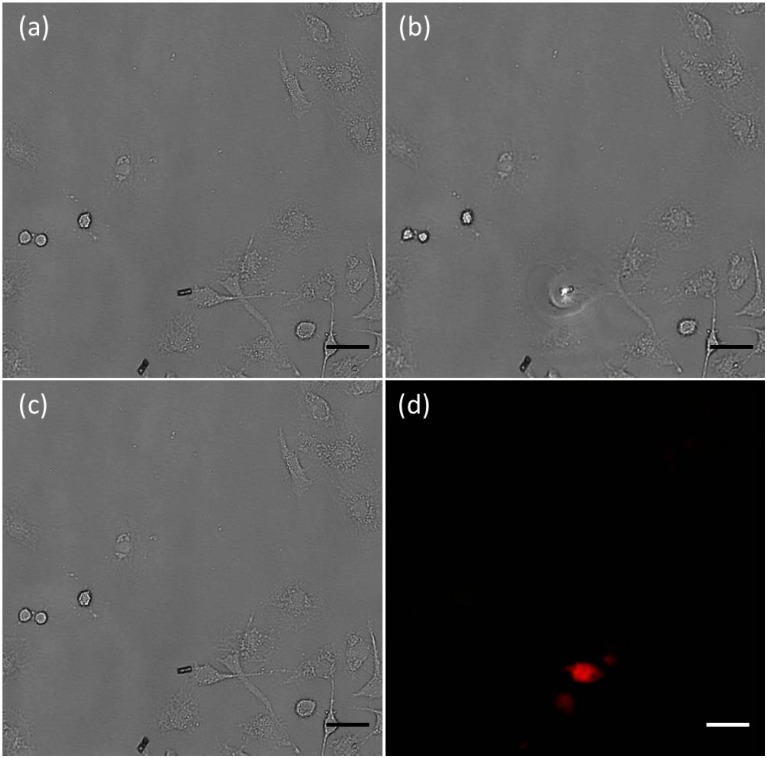
The target photothermal effect of the Au NPs-(PGA/SPI)_14.5_-Fe_3_O_4_ NPs micro-tube to the viability of HeLa cells when under NIR treatment. (**a**–**c**) Time-lapse images illustrating the target movement of the Au NPs-(PGA/SPI)_14.5_-Fe_3_O_4_ NPs micro-tube to the HeLa cells directed by the external magnetic field; (**d**) This photograph shows the corresponding fluorescent image (**c**) of the micro-tube after the radiation of the NIR. Scale bar = 20 μm.

The solution of PI was added into the cell culture to confirm the apoptosis of the HeLa cells. The fluorescence image (Figure 6d) was taken when the micro-tube located to the HeLa cells (Figure 6c) after NIR radiation for one minute and PI staining for 10 min. The image exhibits the red fluorescence from the nuclei of the HeLa cells surrounding the micro-tubes. In contrast, no red fluorescence was observed at approximately 50 μm from the micro-tube where the photo-thermal effect was negligible. The red fluorescence is from the interaction of PI and nucleic acid. PI can penetrate into the nuclei from the breakage of cell membrane and such method has been widely used to evaluate apoptosis of cells. Figure 6d shows the red fluorescence from the nuclei of the cells surrounding the microtube verify distinct apoptotic feature of cells, indicating the photothermal effect of the micro-tubes, under radiation, induced apoptosis of the HeLa cells. When guided by the external magnetic field, the micro-tube induced apoptosis of the HeLa cells near the target location. This outcome, under the combined treatment of magnetic guidance and the irradiation of the NIR, confirmed the target photo-thermal therapy on the cancer cells. Moreover, considered the biodegradable property of the Au NPs-(PGA/SPI)_14.5_-Fe_3_O_4_ NPs micro-tubes, the multifunctional micro-tubes will be degraded by enzymes after their photo-thermal performance, suggesting promising potential application *in vivo*.

## 3. Experimental

### 3.1. Materials

Commercially available porous polycarbonate (PC) membranes with a pore diameter of 5 μm were purchased from the Whatman Corporation-United Kingdom. The Polyethyleneimine (*M*_W_ = 70,000 Da; 50 wt % solution in water) and the Propidium Iodide (PI) were purchased from Sigma-Aldrich Shanghai Trading Co., Ltd., Shanghai, China. The soybean protein isolate (SPI), containing 86.7% protein, was purchased from Gushen Biological Technology Group Co., Ltd., Shandong, China. The Pancreatin powder was obtained from Yakanglinuo Bioengineering Co., Ltd., Jinan, China. The HAuCl_4_·4H_2_O, FeCl_2_·4H_2_O, FeCl_3_·4H_2_O, citric acid monohydrate, NaCl, CH_2_Cl_2_, and ethanol were used without further purification. The water used in all experiments was prepared in a Milli-Q purification system with the resistivity higher than 18.2 MΩ·cm^−1^.

### 3.2. Preparation of Citrate-Stabilized Fe_3_O_4_ Nanoparticles

The citrate stabilized Fe_3_O_4_ NPs were prepared according to the method of Sahoo *et al.* [16]. Briefly, 1.40 g FeCl_3_·4H_2_O and 0.86 g FeCl_2_·4H_2_O were dissolved in 50 mL of distilled water degassed by nitrogen gas. The solution was vigorously stirred by the Stirring Hotplates (Thermal Scientific, Waltham, MA, USA) and heated, under nitrogen protection, for 30 min to 80 °C; then, 8 mL of NH_4_OH was added to the solution, this solution then turned black and was heated for another 30 min. The Fe_3_O_4_ nanoparticles were collected by a magnet and were then rinsed three times. The citric acid solution (4 mL, 0.24 g/mL) was then added to the solution, and the solution was then heated to 95 °C, once again under nitrogen protection. The reaction solution was then cooled to room temperature, while still under nitrogen protection. The Fe_3_O_4_ nanoparticles were collected by a magnet and were rinsed three times using distilled water. The Fe_3_O_4_ nanoparticles suspension was degassed by nitrogen and kept at 4 °C.

### 3.3. Preparation of Citrate Coated Au Nanoparticles

To prepare the citrus-coated Au-nanoparticles a solution of 0.5 mL of 4.0 × 10^−^^2^ M HAuCl_4_ was added to 100 mL of distilled water, followed by the addition of 1 mL of 5.0 × 10^−^^2^ M Trisodium citrate. After waiting five min, 0.6 mL of ice cold 0.1 M NaBH_4_ was added to this solution, this solution was then vigorously stirred for 10 min by the Stirring Hotplates. The color of the solution gradually turned red to indicate the formation of the gold nanoparticles. The Au NPs solution was kept at 4 °C before experimentation [17].

### 3.4. Preparation of Soybean Protein Isolate Micro-Tube

The framework of the soybean protein isolate micro-tubes was prepared through the integration of the PGA and the SPI to the PC membrane [18]. Prior to use, the PGA solution (1 mg/mL in 0.5 M NaCl), and the soybean protein isolated solution (1 mg/mL in PBS solution with pH of 7.2) together with the Fe_3_O_4_ NPs solution were filtered with a 0.45 μm filter. The PC membrane in conjunction with the template was alternatively immersed into the positive charged PGA solution and the negative charged soybean protein solution for one hour as one bilayer. The immediate rinsing step, before immersing into the polyelectrolyte solution, was performed three times. When the 14.5 bilayers of soybean protein were isolated and the PGA was deposited into the template, the template was then immersed into the Fe_3_O_4_ NPs solution for one hour. These components adsorbed both surfaces of the template and were later removed by using wet cotton swabs. The micro-tubes were obtained by dissolving the templates with CH_2_Cl_2_. The micro-tubes were then collected by external magnetic field and rinsed in ethanol and water. 

The assembly of Au NPs to the outer surfaces of the soybean protein isolate micro-tubes was performed by incubating the micro-tubes with citric-stabilized Au NPs for one hour at an approximate room temperature of 27 °C. The micro-tubes were separated using an external magnetic field. The micro-tubes, in solution, performed and then directed movement to the external magnetic field. The movement behavior was recorded by microscope.

### 3.5. Characterization of the Resulting Micro-Tube

A Hitachi S-4300 instrument using an operating voltage of 10 keV conducted the SEM and EDX analysis. For an examination of the micro-tubes, a droplet of each of the micro-tube’s solution was trickled on to a silicon wafer. The TEM images were conducted by using a Tecnai G2 F30 microscope operated at 120 kV. The micro-tube samples were prepared on a carbon-coated copper grid. An Olympus BX53F optical microscope recorded the movement of the micro-tubes and the photothermal effect on the HeLa cells.

### 3.6. Cell Culture

The HeLa cells were incubated in a DMEM medium containing 10% fetal calf serum and 1% penicillin and streptomycin at 5% CO_2_: with humidity. The HeLa cells were divided into a 60 mm polystyrene Petri dish using a standard trypsin-based technique. After the cells were cultivated in the logarithmic growth phase, the micro-tubes were added to the cell culture [19].

### 3.7. Degradable Behavior of the Micro-Tube

The micro-tubes were exposed to α-chymotrypsin solution (5 U/mL) in a phosphate buffer solution (PBS) with a pH 7.4 at room temperature overnight. In the control experiment, the Au NPs-(PGA/SPI) _14.5_-Fe_3_O_4_ NPs micro-tubes were incubated in a plain PBS with a pH of 7.4 at a room temperature of approximately 27 °C. For SEM characterization, the samples were removed from the incubation solution and washed three times.

### 3.8. Analysis of the Photo-Thermal Effort of Micro-Tube under the Irradiation of NIR

The cell culture containing the micro-tubes were irradiated for one minute by using a continuous-wave diode NIR laser (Wuhan Huaray Precision Laser Co., Ltd., Wuhan, China) with a center wavelength of 808 ± 10 nm and output power of 1–5 W. Following the irradiation of the NIR, the viability of the HeLa cells was assessed by performing an apoptosis assay by adding 5 μL of the PI solution to the cell culture. After staining the HeLa cells for 20 min by using Propidium iodide (PI), the fluorescent image of the HeLa cell was taken by an Olympus BX53F optical microscope with an objective of 20× and was then used to confirm the apoptosis of the HeLa cells [20].

## 4. Conclusions

This experiment has described a Fe_3_O_4_ NPs and Au NPs functionalized biodegradable micro-tube based on the integration of polymers to a porous template. The size and geometry of each micro-tube was dependent on the template. The locations of the magnetic micro-tubes were controlled by applying an external magnetic field. Of interest, when under-exposed to the NIR, the Au NPs, on the outer surfaces of the micro-tubes converted the light energy to a heat source, this photo-thermal effect elevated the temperature of the aqueous suspension beyond 42 °C. In this instance, and when under the radiation of the NIR, the micro-tubes located near the cancer cells were able to induce the apoptosis of the HeLa cells. Furthermore, due to the composition of the construct of the micro-tubes, the SPI enable the micro-tubes to degrade by the enzymatic treatment. As the similar photothermal agent was applied *in vivo* [21], the biodegradable and multifunctional micro-tubes have great potential for remotely controlling photothermal therapy, and more deep studies *in vivo* are being conducted.
